# SegAnnDB: interactive Web-based genomic segmentation

**DOI:** 10.1093/bioinformatics/btu072

**Published:** 2014-02-03

**Authors:** Toby D. Hocking, Valentina Boeva, Guillem Rigaill, Gudrun Schleiermacher, Isabelle Janoueix-Lerosey, Olivier Delattre, Wilfrid Richer, Franck Bourdeaut, Miyuki Suguro, Masao Seto, Francis Bach, Jean-Philippe Vert

**Affiliations:** ^1^Department of Computer Science, Tokyo Institute of Technology, Tokyo 152-8552, Japan, ^2^Institut Curie, 26 rue d’Ulm, 75248 Paris Cedex 05, ^3^INSERM U900, Paris F-75248, France, ^4^Mines ParisTech, Centre for Computational Biology, 77300 Fontainebleau, ^5^Unité de Recherche en Génomique Végétale INRA-CNRS-Université d’Evry Val d’Essonne, Évry 91057, France, ^6^INSERM U830, Paris F-75248, France, ^7^Aichi Cancer Center Research Institute, 1-1 Kanokoden, Chikusa-ku, Nagoya-city 464-8681, Japan and ^8^INRIA–Sierra Project-Team, Département d’Informatique de l’École Normale Supérieure, Paris F-75013, France

## Abstract

**Motivation:** DNA copy number profiles characterize regions of chromosome gains, losses and breakpoints in tumor genomes. Although many models have been proposed to detect these alterations, it is not clear which model is appropriate before visual inspection the signal, noise and models for a particular profile.

**Results:** We propose SegAnnDB, a Web-based computer vision system for genomic segmentation: first, visually inspect the profiles and manually annotate altered regions, then SegAnnDB determines the precise alteration locations using a mathematical model of the data and annotations. SegAnnDB facilitates collaboration between biologists and bioinformaticians, and uses the University of California, Santa Cruz genome browser to visualize copy number alterations alongside known genes.

**Availability and implementation:** The breakpoints project on INRIA GForge hosts the source code, an Amazon Machine Image can be launched and a demonstration Web site is http://bioviz.rocq.inria.fr.

**Contact:** toby@sg.cs.titech.ac.jp

**Supplementary information:** Supplementary data are available at *Bioinformatics* online.

## 1 INTRODUCTION

DNA copy number alterations (CNAs) are amplifications, gains and losses of chromosomal regions that can result from different cellular mechanisms, and are important in the study of many types of cancer (Weinberg, 2006). Genome-wide assays such as array comparative genomic hybridization (aCGH) and single nucleotide polymorphism microarrays can be used to detect CNAs. After spatial and sample normalization, these assays yield noisy measurements of approximate copy number with a resolution of up to 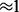 kb between probes.

The goal in analyzing these data is to accurately extract a list of altered regions from each noisy sample. In this article, we define accuracy in terms of annotated regions given by an expert. We treat this expert as a gold standard, and so the goal of our model is to be consistent with his or her annotations. Hocking *et al.* (2013) observed large annotation error rates for several segmentation algorithms applied to a large database of neuroblastoma tumors. In this article, we propose to eliminate these errors with SegAnnDB, a computer vision system whose model always agrees with the provided annotations.

Previous work in DNA copy number analysis can be roughly divided into two categories of methods: visualization and mathematical models. This article combines these two lines of research by proposing a mathematical model that can be iteratively improved by adding visual annotations to zoomed scatterplots of the data. First, we will review previous methods in both categories.

Many software packages have been developed for visualization of aCGH data. For example, Visualization and Analysis of Molecular Profiles (VAMP) can be used for exploratory analysis, or to visualize predicted alterations from a model (La Rosa *et al.*, 2006). Another visual analysis program is ChARMView, which allows manual identification of regions for significance testing (Myers *et al.*, 2005). A potential problem with these programs is that the displayed model is calculated before visualization, and cannot be interactively updated.

Several Web sites for array CGH analysis have been proposed. CGHweb allows visual comparison of several algorithms applied to the same normalized profile (Lai *et al.*, 2008). ArrayCyGHt and CAPweb provide normalization and copy number calling (Kim *et al.*, 2005; Liva *et al.*, 2006). ISACGH supports analysis, segmentation, visualization and export to the Ensembl genome browser (Conde *et al.*, 2007; Flicek *et al.*, 2012). ArrayFusion exports data and segmentations in formats suitable for genome browsers (Yang *et al.*, 2006). Like the method we propose in this article, these Web sites facilitate collaboration with biologists. Unlike these Web sites, our SegAnnDB software allows the user to interactively update the displayed segmentation model.

In contrast to visual methods for alteration detection, mathematical models can be used to automatically predict lists of alterations based on certain mathematical assumptions about the data. The available mathematical models specifically designed for detecting CNAs are summarized by Neuvial *et al.* (2011). However, a major problem with this class of methods is model selection. Given a particular dataset to analyze, it is obvious to choose neither a particular model nor its tuning parameters. How to evaluate which model is best?

Without doing more experiments, the only method of evaluation is to plot the model alongside scatterplots of the data. A good model should capture all visible alterations in the data and should not predict any false-positive detections. This visual criterion for model evaluation can be used by creating a database of annotated regions that encode an expert’s interpretation of the data (Hocking *et al.*, 2013). In that study, default parameter values of several models were shown to yield many false-positive and false-negative detections. And even after tuning the parameters of each model, there were no models with perfect detection accuracy.

**Fig. 1.**
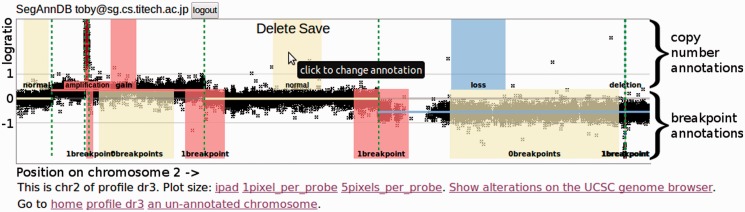
General workflow in annotation-guided DNA copy number analysis on SegAnnDB. Scatterplots of black points show log ratio as a function of genomic position. Breakpoints in the current segmentation are shown with vertical dashed lines, and predicted copy number status of each horizontal line segment is indicated by its color (Supplementary Table S2). Annotated regions can be added to update the copy number (top) and breakpoints (bottom) in the displayed model. Dragging on an unannotated region creates a new region with Save and Delete buttons, as shown for the normal region in the center. Before saving, the annotation can be changed by clicking the region. After saving, the displayed model is immediately updated to agree with the annotation

In this article, we propose to solve this problem by interactively annotating alterations in scatterplots of the data (Fig. 1). Then our software selects a tuning parameter that agrees with the user-defined annotations and immediately shows the updated model. If the displayed segmentation model does not capture the alterations that are obvious from visual inspection, then annotations can be added to correct the model. A user-specific model can thus be iteratively improved until it agrees with the annotator’s visual interpretation of the data.

In addition, the annotations can be assembled into a database, so we can apply algorithms that automatically recognize previously annotated patterns. We note that this computer vision approach has also been successful for recognizing phenotypes in cell microscopy (Jones *et al.*, 2009). After annotating a small subset of the data, the system adapts to the annotations and provides consistent predictions for unannotated data.

This article describes SegAnnDB, a Web-based free/open-source implementation of this interactive genomic segmentation model. The name is short for Segmentation and Annotation DataBase, as annotations are stored in a database, which is used by machine learning algorithms to find an appropriate segmentation. After interactive annotation, the learned segmentation model can be directly exported to the University of California, Santa Cruz (UCSC) genome browser for viewing detected alterations alongside known genes (Kent *et al.*, 2002). 

**Fig. 2.**
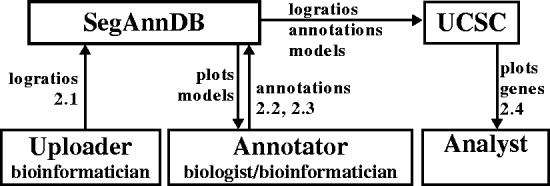
SegAnnDB exports data to the UCSC genome browser and facilitates collaboration between bioinformaticians and biologists. First, profile probe log ratio values are uploaded to a SegAnnDB server by an uploader (Section 2.1). Then an annotator can plot the data and refine a segmentation model by adding annotations (Sections 2.2 and 2.3). Finally, an analyst can send data to the UCSC genome browser, which displays plots of the segmentation model with known genes (Section 2.4)

Finally, SegAnnDB promotes collaboration between biologists, doctors and bioinformaticians doing genome-wide copy number analysis (Fig. 2). Collaboration using SegAnnDB is simple: once a bioinformatician uploads a profile, anyone with a Web browser can create a user-specific segmentation model by drawing annotated regions on the scatterplots (Fig. 1). This makes it easy for people with expert prior knowledge but no programming experience to browse the profiles and annotate regions of interest (e.g. a biologist looking for alterations in known oncogenes). After annotation, the bioinformatician can download the annotations and segmentation model for further analyses such as detection of common alterations in several related samples, or survival regression based on the detected alterations.

## 2 SYSTEM AND METHODS

In this section, we describe the general workflow when using a SegAnnDB server for annotation-guided DNA copy number analysis. Later in Sections 3 and 4, we give details about how the server interactively calculates and displays the models.

### 2.1 Uploading profiles

The first step of any analysis is to upload the normalized log ratio data to the Web site. The data should be uploaded in gzipped UCSC bedGraph format, as this is the format that will also be used to export the data for viewing on the UCSC genome browser (Kent *et al.*, 2002). The four-column text-based bedGraph format is simple, so any bioinformatician should be able to quickly convert data from any platform-specific format.

The bedGraph header line must contain the following three important variables specific to SegAnnDB. The db variable should indicate the genome version of the probe positions (e.g. db=hg19). The maxSegments variable specifies the maximum number of segments per chromosome for the initial SegAnnDB scatterplots, before manual annotation (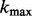 in Section 3.1). The share variable controls who can view the profile:

- share=public means all users of the Web site.
- A domain name such as share=stat.berkeley.edu or share=curie.fr means only users with emails from the indicated domain.
- share=private means only the user who uploaded it.

Profiles can be uploaded one at a time using the Web upload form or several at a time by using the upload_profiles.py command line program. Once a profile has been uploaded, it must undergo two main processing steps before interactive annotation is possible. First, the log ratio values and several maximum likelihood segmentation models are saved in BerkeleyDB, a highly efficient database system. Second, Portable Network Graphics (PNG) scatterplots are generated for several zoom levels (Supplementary Table S1). The only limit on the number of profiles is the amount of disk space available on the SegAnnDB server. Importantly, the amount of time and disk space required is linear in the profile size (Table 1).

**Table 1.** System requirements for profiles of different sizes

Profile size probes	Time seconds	BerkeleyDB megabytes	Scatterplots megabytes	Probes.gz megabytes
2678	1	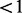	1	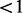
56 855	3	1	1	1
262 230	10	3	3	3
1 868 857	100	22	27	28

*Note*: The processing time includes calculating segmentation models and PNG scatterplots on a 2.9 GHz Intel i7-3520 M CPU. We show disk space occupied by a profile in the database (BerkeleyDB), the total size of the PNG images (Scatterplots) and the size of the data file to upload and export to UCSC (Probes.gz).

### 2.2 Plotting data and annotating breakpoints

Once a profile has been uploaded and processed, it can be plotted and annotated. From the home page or the list of profiles, clicking a profile name shows a zoomed out plot of all its chromosomes. Plots can be zoomed to individual chromosomes and then zoomed further by clicking the plot size links shown in Figure 1.

Each plot initially shows the uploaded data as black points and a segmentation model as horizontal line segments (Fig. 1). We use vertical dashed lines to draw the ‘breakpoints’, which are the change points in the piecewise constant segmentation model.

The breakpoints in the displayed segmentation can be edited by adding breakpoint annotations to the bottom half of the plot. There are two types of breakpoint annotations: 1breakpoint means there is exactly one breakpoint in the region, and 0breakpoints means there are no breakpoints in the region. Dragging on an unannotated region creates a new region with Save and Delete buttons, as shown in Figure 1. Before saving, an annotation can be changed from 1breakpoints to 0breakpoints by clicking the region. After saving the annotation, it is sent to the server, which calculates the consistent segmentation model defined in Section 3.1. The server immediately sends the consistent segmentation back for display in the Web browser. Thus, the segmentation model can be iteratively updated by adding breakpoint annotations, until the displayed breakpoint locations match the expert interpretation of the annotator.

As explained in Section 3.1, one of the two possible segmentation models will be used: pruned dynamic programming (PrunedDP) or SegAnnot. The color of the dashed vertical breakpoint lines indicates the algorithm: green for PrunedDP and purple for SegAnnot. It is important to note that when the SegAnnot algorithm is used, there must be a 1breakpoint annotation for each breakpoint in the segmentation. In contrast, the PrunedDP algorithm can detect breakpoints in unannotated regions.

### 2.3 Annotating copy number

After finding a model with appropriate breakpoints, copy number annotations can be added to the top half of each plot to define copy number status (Fig. 1). Each copy number annotation should define a region of equal copy number: deletion, loss, normal, gain or amplification (Supplementary Table S2). More types of copy number annotations can be defined for specific projects by editing the SegAnnDB source code. After adding or deleting a copy number annotation, SegAnnDB updates the predicted copy number status of each segment of the profile. This is indicated by immediately updating the color of the displayed horizontal line segments. This immediate genome-wide visual feedback is crucial for avoiding accidental mistakes in annotation.

There are two special colors for horizontal line segments: green and black. If a profile has no copy number annotations, then all segments are colored green. If a segment has several different overlapping copy number annotations, it is colored black to indicate that it should be corrected.

Copy number status is generalized across segments of a profile, so not all segments need to be labeled. The segments with overlapping copy number annotations are used to infer the copy number status of the other unlabeled segments. However, one copy number annotation of each type must be present to use this feature. For example, if there is only one normal and one gain annotation on a profile, then the rest of the segments will be labeled as either normal or gain, and there will be no segments labeled loss.

### 2.4 Exporting data to the UCSC genome browser

**Fig. 3.**
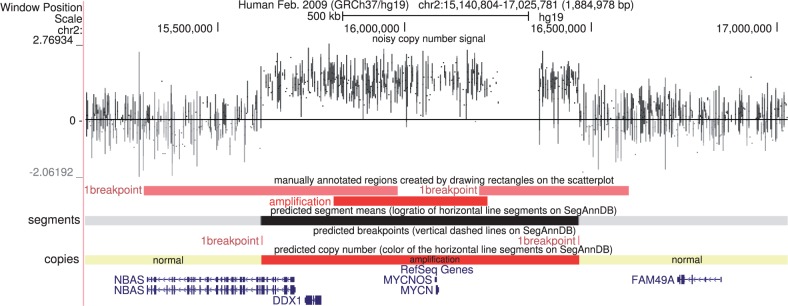
The data, annotated regions and labeled segments are exported for visualization alongside RefSeq genes on the UCSC genome browser. From top to bottom, the probe log ratios are shown in black, the annotated regions are shown using their colors on SegAnnDB, the log ratio of the segmentation model is shown using a white 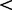 black scale, the breakpoints in the segmentation model are red and the inferred copy number status of each segment is shown using the copy number annotation colors of Supplementary Table S2. In this example, it is clear that the MYCN oncogene is amplified in this neuroblastoma tumor sample

After a profile has been appropriately annotated, a table of detected alterations can be displayed by clicking the ‘Show alterations on the UCSC genome browser’ link shown on the bottom of Figure 1. Clicking a button on that page exports the probes, annotations and segmentation to the UCSC genome browser (Fig. 2). Although positions of genes can not be viewed while annotating profiles on SegAnnDB, they can be viewed alongside the exported segmentation model on the UCSC genome browser (Fig. 3). Data from multiple profiles can be displayed at the same time on UCSC, for rapid visual verification of repeated alterations in particular genes.

There are two methods for quickly navigating from SegAnnDB to altered regions on the UCSC genome browser. First, shift-clicking any annotated region opens a new Web page with the UCSC genome browser zoomed to that region. Second, each profile has an alterations table that contains links to each detected breakpoint, gain, loss, amplification and deletion.

The annotations and displayed segmentation models can also be downloaded for other analyses. For example, the annotations could be used to develop better models for breakpoint detection or copy number calling. Also, the displayed segmentation model could be used for further analyses such as survival regression or plotting a genome alteration print (Popova *et al.*, 2009).

## 3 ALGORITHM

In this section, we explain the algorithmic details of the models used to detect breakpoints and CNAs.

### 3.1 Calculating the displayed segmentation model

To find an appropriate segmentation model for each chromosome, we first calculate a sequence of segmentation models, then use the expert’s breakpoint annotations to choose a consistent model.

Let 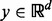 be the normalized log ratios for one chromosome, measured at positions 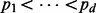. This signal is drawn using black points in Figure 1. Because of its speed and breakpoint detection accuracy (Hocking *et al.*, 2013), we segment using the PrunedDP algorithm of Rigaill (2010). It calculates the least squares segmentation 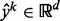 for every 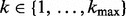 segments:
(1)
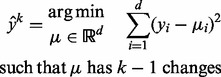



The PrunedDP algorithm returns a sequence of progressively more complex segmentation models 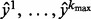. For each model size 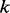, the segmentation 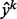 has the least squared error among all models with 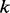 segments.

The maximum number of segments 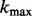 is the only parameter of PrunedDP. For interactive annotation on the SegAnnDB Web site, we usually set 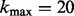, which means that up to 20 segments will be shown on the initial scatterplot, before manual annotation. After annotation, if there are ≥20 breakpoint annotations, then SegAnnot will be used instead of PrunedDP, as explained below. For high-density arrays with many expected breakpoints (e.g. chromothripsis), larger values may be specified (e.g. 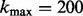). However, 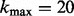 was a reasonable choice for even the high-density arrays that we analyzed (Supplementary Fig. S1 and Supplementary Table S3).

The model selection problem is to choose one of the 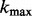 segmentation models, which we do using breakpoint annotations. Let 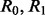 be the sets of 0breakpoints and 1breakpoint annotations, respectively. These appear on the bottom half of the scatterplots shown on SegAnnDB (Fig. 1). Each 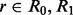 is an interval of base pairs, so to compare with the segmentation model, we need to convert the model breakpoint locations to base pairs. For each model size 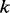, we estimate the set of base pairs after which a change occurs using
(2)
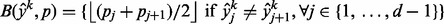



Equation (2) defines a breakpoint for every change 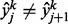 in the model, at the base pair halfway between the probes 
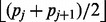
. These breakpoint positions are shown using dashed vertical lines on SegAnnDB scatterplots (Fig. 1).

We calculate the annotation error to determine which PrunedDP models agree with the set of current breakpoint annotations. The annotation error 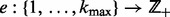 uses the zero–one loss to judge agreement of the predicted breakpoints 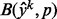 and the annotated regions 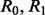:
(3)
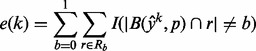



The indicator function 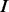 is 0 when the model 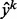 predicts the correct number of breakpoints in a region 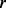, and 1 otherwise. If the model with 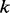 segments has no annotation error 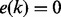, then we say that the segmentation 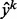 is consistent with the given annotations. The set of consistent PrunedDP models is 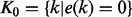.

**Fig. 4.**
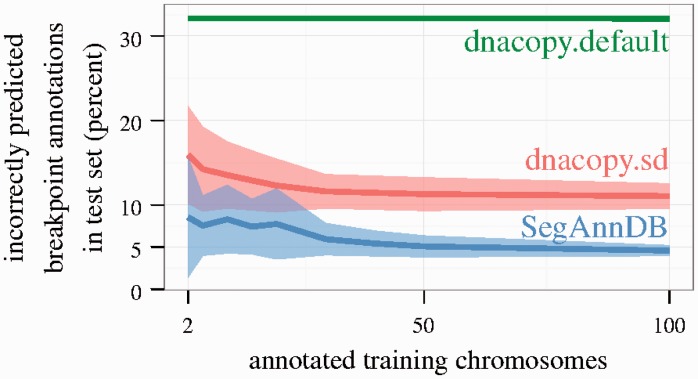
Cross-validation was used to estimate breakpoint detection error in the neuroblastoma.U830.bac dataset. It is clear that the supervised methods (dnacopy.sd, SegAnnDB) adapt to the training set, and provide better breakpoint detection on test data. Note that dnacopy.sd sometimes had lower test error than SegAnnDB in the other datasets we examined (Supplementary Fig. S5)

If there are any consistent PrunedDP models, then 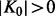 and we define the optimal number of segments as
(4)
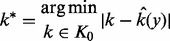

where the predicted number of segments 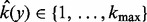 is learned using the log.s.log.d max margin interval regression model on the other annotated chromosomes (Rigaill *et al.*, 2013). In short, 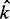 is a prediction function that minimizes the average annotation error over all annotated chromosomes. So, as annotations are added, the predicted set of breakpoints tends to get more accurate (Fig. 4). To support fast model updates during interactive annotation, the prediction function 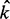 is learned in the background on the Web server. Equation (4) is used to pick the consistent model 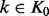, which is closest to the complexity of the predicted model 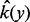.

If there are no consistent PrunedDP models then 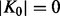 and we use the SegAnnot dynamic programming algorithm (Hocking and Rigaill, 2012). SegAnnot exactly recovers the least squares segmentation such that the annotation error is 0, meaning that each 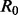 region has 0 breakpoints and each 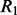 region has exactly 1 breakpoint:
(5)
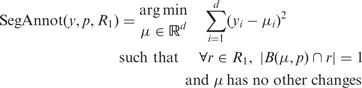



The constraint in (5) means that the estimated mean 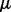 changes once in each 1breakpoint region 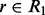, and has no changes elsewhere.

In summary, the displayed segmentation is given by
(6)
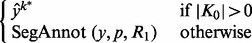



By construction, this segmentation is consistent with the breakpoint annotations 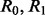.

### 3.2 Calculating copy number state

Segments with overlapping copy number annotations are used to infer the copy number status of unannotated segments on the same profile. Copy number status is inferred by learning a set of up to four thresholds, between the five possible copy number annotations (Supplementary Table S2). Each of these thresholds is learned in the same way, which we explain below for just the threshold between normal and gain.

Let 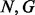 be the sets of normal and gain segment means, respectively. Let there be 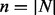 normal segments and 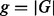 gain segments, and let 
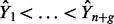
 be the ordered segment means. We consider thresholds 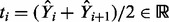 for all 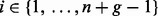. Given a threshold and a segment mean 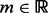, we predict copy number status
(7)
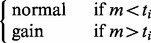



The best threshold minimizes the number of incorrectly predicted copy number annotations:
(8)
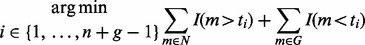



The indicator functions 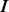 are 0 when the threshold 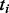 correctly predicts the copy number status of an annotated segment, and 1 otherwise. The best threshold (8) can be calculated in linear 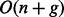 time.

## 4 IMPLEMENTATION

In this section, we discuss the technologies required to implement a Web-based interactive system like SegAnnDB.

### 4.1 Interactive scatterplots

Scatterplots of probe log ratio values are displayed quickly on SegAnnDB, as they are pre-rendered and saved as bitmap PNG images when a profile is uploaded. Some zoom levels are scaled in proportion to the number of probes, so high-density profiles can result in large PNG images (Supplementary Table S1). Some Web browsers do not render these large PNG images. For example, current iPad Web browsers do not render images wider than 20 000 pixels, so we defined an “ipad” zoom level specifically to accommodate this maximum possible zoom level. Among desktop Web browsers, we found that Google Chrome works best on several platforms.

Interactive model updates are implemented using the D3 Javascript library (Bostock *et al.*, 2011). The basic idea is to draw the segmentation model and breakpoints for each chromosome on an HTML Web page with an SVG element that has the PNG scatterplot as the background. After each change to the annotated regions, the server updates the displayed model by sending a JavaScript Object Notation file to the annotator’s Web browser.

### 4.2 Data storage, export and server configuration

To support interactive annotation and model updates, SegAnnDB needs to store data in a fast database system. We used Berkeley DB, as it allows quick storage and retrieval of any data types. In addition, the disk space requirements are approximately linear in profile size (Table 1).

Data can be exported to the UCSC genome browser by clicking a button on the SegAnnDB Web site. This sends the copy number data, annotations and model directly from SegAnnDB to the UCSC genome browser. Figure 2 explains the relationship between the uploader, annotator, SegAnnDB server and UCSC genome browser.

Configuring the SegAnnDB software on a Debian/Ubuntu GNU/Linux system requires Python and several free/open-source extension packages. For example, the Pyramid Web framework and the SegAnnot and PrunedDP segmentation modules are required. To avoid the time-consuming task of configuring all these packages for every new SegAnnDB server, we made a public Amazon Machine Image, so launching a new SegAnnDB server takes only 10 min on Amazon’s Elastic Compute Cloud. A list of current Amazon Machine Images can be found in the SegAnnDB source code README on INRIA GForge.

## 5 RESULTS AND DISCUSSION

### 5.1 Supervised versus unsupervised analysis

In the machine learning literature, a learning problem is ‘supervised’ when there is a teacher or oracle that provides correct predictions for training an algorithm. In this article, the type of supervision that we proposed is a set of annotated regions from an expert’s visual interpretation of the data. These annotated regions are then used by SegAnnDB to build a model with consistent breakpoint locations and copy number calls.

In contrast, most statistical models for DNA copy number analysis can be viewed as unsupervised because they do not use an annotation database for model training. Typically, an unsupervised statistical model is first fit to the data, and then an expert plots the model to judge if it fits the data well. This sequence of steps is inverted on SegAnnDB: first, we plot and annotate the data, and then we fit a model to the combined data and annotations.

We considered the DNAcopy circular binary segmentation model of Venkatraman and Olshen (2007) as an unsupervised baseline model and compared its performance with SegAnnDB. We analyzed speed, train error and test error with respect to seven annotation datasets, consisting of 708 neuroblastoma, lymphoma and medulloblastoma copy number profiles (Supplementary Table S4). The data come from cell lines and primary tumors (Supplementary Fig. S2) analyzed using different platforms (Bacterial Artificial Chromosome (BAC)/P1 Artificial Chromosome (PAC), Nimblegen, Affymetrix), so the number of probes per profile ranges from 1719 to 1 868 857 (Supplementary Table S3). In total, there were 4467 annotated chromosomes containing 6937 annotated regions.

Supplementary Table S5 shows that the PrunedDP algorithm used by SegAnnDB took <2 h to train on the entire dataset, but DNAcopy took >3 h for each of the 31 parameter values we tested. PrunedDP was faster overall because it was faster for the high-density profiles (Supplementary Fig. S3).

The displayed segmentation on SegAnnDB is always consistent with the given breakpoint annotations, so SegAnnDB has 0% training error by definition (Section 3). In contrast, it may be impossible to achieve zero annotation errors with an unsupervised model that does not directly use the breakpoint annotations (Hocking and Rigaill, 2012). Supplementary Table S6 shows that the unsupervised dnacopy.default algorithm predicts 4–50% incorrect breakpoint annotations across the seven datasets we examined. For a more balanced comparison, Hocking *et al.* (2013) showed that DNAcopy breakpoint detection can be improved by picking an undo.SD parameter which minimizes breakpoint annotation error (we did not apply smoothing before segmentation, and we kept default values for all other parameters). For each dataset, we picked chromosome-specific undo.SD values, yielding better training error rates (Supplementary Table S6, dnacopy.sd, 0.33–2.75%). Although breakpoints detected by these methods are qualitatively similar (Supplementary Fig. S4), SegAnnDB is quantitatively more accurate with 0% error.

We also evaluated the test error by training a model on a set of annotated chromosomes, and counting the number of incorrectly predicted breakpoint annotations on a test set. Figure 4 and Supplementary Figure S5 show that adding more annotations improves the test error of the supervised SegAnnDB and dnacopy.sd methods (dataset-specific parameters were chosen by minimizing annotation error for each randomly chosen training set). Predictably, the supervised methods adapt to the provided annotations and detect breakpoints more accurately than the unsupervised dnacopy.default algorithm. However, on some high-density datasets, even the best methods showed up to 20–40% test error (Supplementary Fig. S5). The large test error of these supervised methods motivates spending time on interactive annotation, which always achieves 0% training error.

SegAnnDB is a computer vision system that exploits the strong points of the human visual system and mathematical models. The human visual system is good at detecting noise and breakpoints in the copy number signal over large regions, but bad at detecting the precise location of a breakpoint. Mathematical models are good at detecting the precise locations of alterations, if tuning parameters are adjusted appropriately. SegAnnDB combines the best of both approaches by automatically adjusting the tuning parameters of a mathematical model to agree with an expert’s visual annotations.

### 5.2 Time required for visual annotation on SegAnnDB

The amount of time is proportional to the number of annotated regions that need to be added to correct the displayed model. More breakpoint annotations are sometimes necessary for larger profiles (Supplementary Fig. S6), but when SegAnnDB provides good predictions in unannotated regions (Fig. 4), annotating all breakpoints is not necessary (Supplementary Fig. S1). So in our experience, it takes ∼10 min to completely annotate even high-density profiles, and it should be feasible to annotate each profile in datasets of a few dozen samples.

For larger datasets, it may not be feasible to annotate every profile. For example, visually annotating every profile from the 1000 Genomes Project of Altshuler *et al.* (2010) would require ∼10 min/profile 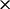 1000 profiles
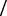
(60 min/h) = 166 h, which is several weeks of work. However, for large datasets, SegAnnDB can still be useful for creating a relatively small database of 5–10 visually annotated profiles that are representative of the bigger dataset (generated on the same platform and for the same tumor type). Then the protocols of Hocking *et al.* (2013) can be used for training and validating an algorithm for automatically detecting CNAs on the unannotated profiles. The main idea is that for each manually annotated region, one can check whether an algorithm predicts the indicated copy number or breakpoints in that region. If there is some disagreement, then the algorithm needs to be improved. So when there is not enough time to visually annotate every profile, an annotated region database is still useful for checking the validity of an automatic annotation algorithm.

### 5.3 Correcting experimental artifacts

Supervised copy number analysis is useful for correcting the various types of noise and artifacts that can be present in DNA copy number profiles. In this section, we discuss three types of corrections that can be easily visually annotated using SegAnnDB.

**Fig. 5.**
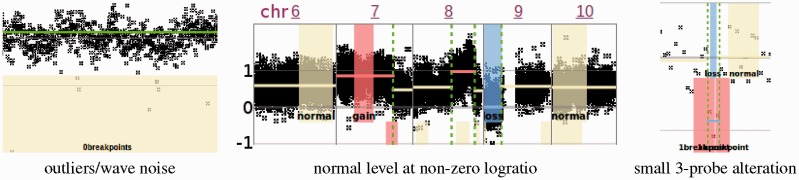
Examples of noise and alterations that can be annotated using SegAnnDB. Left: outliers and wave noise can be ignored using 0breakpoints annotations. Center: normal copy number at a non-zero log ratio value can be indicated using normal annotations. Right: small alterations can be annotated using 1breakpoints and copy number annotations

First, some profiles have noise patterns that should be ignored and are easy to visually identify. Examples are wave patterns and outliers, as shown in the left panel of Figure 5. If one of these patterns can be visually identified, then SegAnnDB can be used to exclude it by simply placing a 0breakpoints region around it.

Second, many copy number calling algorithms assume that the baseline normal level of two copies should be centered around log ratio 0, but this can depend on the normalization procedures. For example, the center panel of Figure 5 shows a profile whose normal level appears at log ratio 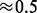. When it is visually obvious that normal copy number is at a non-zero log ratio value, then normal copy number regions can be used to indicate that.

Finally, many algorithms have a parameter for the minimal number of probes required for identifying a CNA. For example, by default DNAcopy sets its min.width parameter to two probes. Instead of defining an arbitrary value that may not hold for all alterations in a dataset, breakpoint annotations can be used to define small alterations that are visually obvious, such as the loss shown in the right panel of Figure 5. Then, for example, the annotations can be used to check which values of the DNAcopy min.width parameter are appropriate for a given dataset.

### 5.4 Using annotations in later analyses

The annotation database, segmentation and copy number calls created on SegAnnDB can be used as inputs to other analyses. SegAnnDB creates a list of breakpoints which is more accurate than other methods such as DNAcopy (Supplementary Table S6), and this degree of accuracy can be critical for several applications.

For example, to construct a genome alteration print, Popova *et al.* (2009) require a good segmentation algorithm: ‘the absence of true breakpoints could significantly alter the GAP pattern’. Annotated regions can be used on SegAnnDB to ensure that the segmentation contains all visible breakpoints.

As another example, the locations of detected alterations can be used to construct predictors when modeling survival outcome. SegAnnDB can be used to construct predictors that agree with an expert’s visual interpretation of the data.

An important final example is detecting recurrent alterations in related samples, or excluding germ line alterations that also appear in normal samples. There are two approaches: either the model shown on SegAnnDB can be post-processed, or the annotated regions created using SegAnnDB can be used to train and validate another algorithm.

## 6 CONCLUSIONS

We described the usage and implementation of SegAnnDB, a Web-based computer vision system for DNA copy number profile analysis. SegAnnDB improves previous visualization approaches by allowing the model to be interactively updated using annotated regions. In contrast with other mathematical models in the literature, SegAnnDB has no tuning parameters, as they are selected automatically using the provided annotations. Overall, SegAnnDB is a useful research tool that provides accurate annotation of copy number profiles, facilitates interaction between biologists and bioinformaticians, and allows visualization of several copy number profiles simultaneously.

Annotated regions can be added on SegAnnDB until the displayed model is consistent with an expert’s visual interpretation of a copy number profile. In other words, an expert with enough time can always find a consistent model by adding annotations, as our model always has zero training error with respect to the annotated regions.

However, predicting accurate breakpoints for unannotated test data was difficult for the models we considered (Supplementary Fig. S5). Developing models that can more quickly achieve better breakpoint detection and copy number calling in unannotated test data remains an interesting research direction. Although different experts do not always provide consistent annotations (Supplementary Fig. S7), we are also investigating a multitask learning model that could potentially have better prediction accuracy for each of those experts.

One current feature of SegAnnDB is the ability to plot a random unannotated chromosome. Instead of plotting a random profile, we could plot a profile that is likely to improve model predictions. We are interested in future research into sampling methods that improve the model faster than random sampling in DNA copy number profile annotation databases.

Finally, we are interested in using visual annotations for modeling copy number changes in next-generation sequencing data. In particular, Teo *et al.* (2012) show figures with clear breakpoints in read depth and fragment count. Boeva *et al.* (2012) show plots with clear breakpoints in copy number and B allele frequency. These breakpoints can be visually annotated and saved to a database to ensure that they are respected by any trained models.

## Supplementary Material

Supplementary Data
