# Supplementary material for: SegAnnDB: interactive Web-based genomic segmentation
Source: Bioinformatics. 2014 Feb 3;30(11):1539–46. doi: 10.1093/bioinformatics/btu072 (PMC4029035; doi:10.1093/bioinformatics/btu072)
Supplement: Supplementary Data [file supp_30_11_1539__index.html]

SegAnnDB: interactive Web-based genomic segmentation — SegAnnDB: interactive Web-based genomic segmentation — Supplementary Data 

# SegAnnDB: interactive Web-based genomic segmentation

## Supplementary Data

files

**Files in this Data Supplement:**

- Supplementary Data - pdf file
