# Supplementary material for: SegAnnDB: interactive Web-based genomic segmentation
Source: Bioinformatics. 2014 Feb 3;30(11):1539–46. doi: 10.1093/bioinformatics/btu072 (PMC4029035; doi:10.1093/bioinformatics/btu072)

Supplementary figures

Toby Dylan Hocking

October 1, 2013

Table S1: Width of scatterplots is constant or scaled by the number of probes

Zoom levels can be defined on a per-server basis, and by default there are four levels for scatterplots of chromosomes. The width in pixels of the PNG scatterplot of chr1 is shown for two different sized profiles (columns) and four zoom levels (rows). The width in pixels of standard and ipad images are the same for profiles of different size, but the other plots are scaled based on the number of probes.

zoom level	398 probes	146,401 probes
standard	1,500	1,500
ipad	20,000	20,000
1pixel_per_probe	398	146,401
5pixels_per_probe	1,990	732,005

Table S2: segment color indicates predicted copy number status

Color code for horizontal line segments, which indicate predicted copy number on SegAnnDB. Black means that several different copy number annotations have been assigned to the same segment, and this conflicting situation should be corrected. Green means that no copy number annotations have been added to the profile.

color		annotation	copy number
dark red		amplification	$\gg 2$
light red		gain	> 2
yellow		normal	≈ 2
light blue		loss	1
dark blue		deletion	0
black		multilabeled	
green		unlabeled	

Table S3: At most 26 annotated breakpoints per chromosome

For each data set, we show the range of probes per profile, probe density, and minimum, interquartile range, mean, and maximum number of 1breakpoint annotations per chromosome. For example, in the lymphoma.aichi.msuguro data set, the chromosome with the most annotated breakpoints had 18 regions annotated as containing 1breakpoint. The mean and median number of annotated breakpoints is from 0 to 3 regions in each data set, and the maximum over all sets is 26. It is clear that there are more annotations for the higher density profiles. In our offline analysis of these data, we set the maximum number of segments parameter $k_{\max} = 52$ (in contrast, we usually set $k_{\max} = 20$ during interactive analysis on the SegAnnDB web site).

	probes/profile		probe density (bases/probe)		range of 1breakpoint annotations per chromosome					
set	min	max	min	max	Min.	1st Qu.	Median	Mean	3rd Qu.	Max.
neuroblastoma.U830.bac	1719	5535	129713	3156280	0	0	0.0	0.16	0	4
neuroblastoma.U830.nimblegen	71341	71341	39949	40139	0	0	0.0	0.04	0	2
lymphoma.aichi.tdh	41687	410696	4102	80896	0	0	0.5	1.00	2	4
lymphoma.aichi.msuguro	41526	410696	4102	81492	0	0	1.0	2.89	5	18
neuroblastoma.chiba	58477	262230	3768	17627	0	0	0.0	1.02	1	23
medulloblastoma.U830	71334	1813281	591	40218	0	0	2.0	1.81	2	25
neuroblastoma.U830.SNP6	1868417	1868571	560	818	0	0	2.0	1.80	2	26

Table S4: Annotation counts for each data set

Counts of annotated profiles, chromosomes, and regions are shown for all data sets. The data come from 3 research groups:

- INSERM U830 coauthors Schleiermacher, Janoueix-Lerosey, Bourdeaut, Richer, Delattre: neuroblastoma and medulloblastoma samples taken from cell lines and primary tumors analyzed at the Institute Curie.
- Aichi cancer center coauthors Suguro and Seto: malignant lymphoma (Diffuse Large B-cell lymphoma), samples originating from the lymph node, peripheral blood, bone marrow, and ascites.
- neuroblastoma.chiba are public data downloaded from NCBI GEO, accession number GSE5784.

Note that the total of the profiles column is 709, but we wrote in the text that there are 708 total profiles. This is because 1 profile in the lymphoma data set was labeled by two different experts (Figure S7).

set	profiles	chroms	regions	1breakpoint	0breakpoints
neuroblastoma.U830.bac	541	3109	3642	485	3157
neuroblastoma.U830.nimblegen	34	182	188	7	181
lymphoma.aichi.tdh	5	44	96	44	52
lymphoma.aichi.msuguro	12	63	261	182	79
neuroblastoma.chiba	25	123	218	125	93
medulloblastoma.U830	59	653	1748	1180	568
neuroblastoma.U830.SNP6	33	298	784	537	247

Table S5: DNAcopy is slower than PrunedDP

We report the number of hours and minutes on a 3GHz Intel Xeon CPU, for calculating the segmentation models for all profiles.

algorithm	models	time
dnacopy.default	1	3h59m
dnacopy.sd	31	127h37m
SegAnnDB (PrunedDP)	52	1h45m

Table S6: SegAnnDB has 0% breakpoint detection training error

Percent breakpoint annotation training error was calculated for all models and data sets. The dnacopy.default algorithm shows many errors, and dnacopy.sd reduces that to a few percent by tuning the undo.SD parameter for each chromosome (keeping defaults for all other parameters). The PrunedDP algorithm is better but still has a few errors, so in those cases SegAnnDB uses SegAnnot, which always has 0% training error by definition (Section 3.1).

set	regions	dnacopy.default	dnacopy.sd	PrunedDP	SegAnnDB (SegAnnot)
neuroblastoma.U830.bac	3642	27.84	0.33	0.03	0
neuroblastoma.U830.nimblegen	188	50.00	0.53	0.53	0
lymphoma.aichi.tdh	96	12.50	1.04	0.00	0
lymphoma.aichi.msuguro	261	3.83	0.38	0.00	0
neuroblastoma.chiba	218	26.61	2.75	0.00	0
medulloblastoma.U830	1748	12.19	0.92	0.00	0
neuroblastoma.U830.SNP6	784	15.18	1.66	0.13	0

Figure S1: SegAnnDB usually predicts more breakpoints than annotated

For each annotated chromosome, we plot the number of segments displayed on SegAnnDB versus the number of 1breakpoint annotations added by the annotator. A bit of noise jitter has been added to each point to avoid overlap. Points on the diagonal line represent chromosomes with as many 1breakpoint annotations as displayed segments. It is clear that SegAnnDB often predicts more breakpoints than annotated, but there are sometimes complicated profiles that require adding many annotations and using SegAnnot (blue dots on the diagonal, e.g. lymphoma.mkatayama). In addition, it is clear that our suggestion of $k_{\max} = 20$ for PrunedDP for interactive analysis is a reasonable choice in these data sets (for our offline analysis in this figure, we used $k_{\max} = 52$).

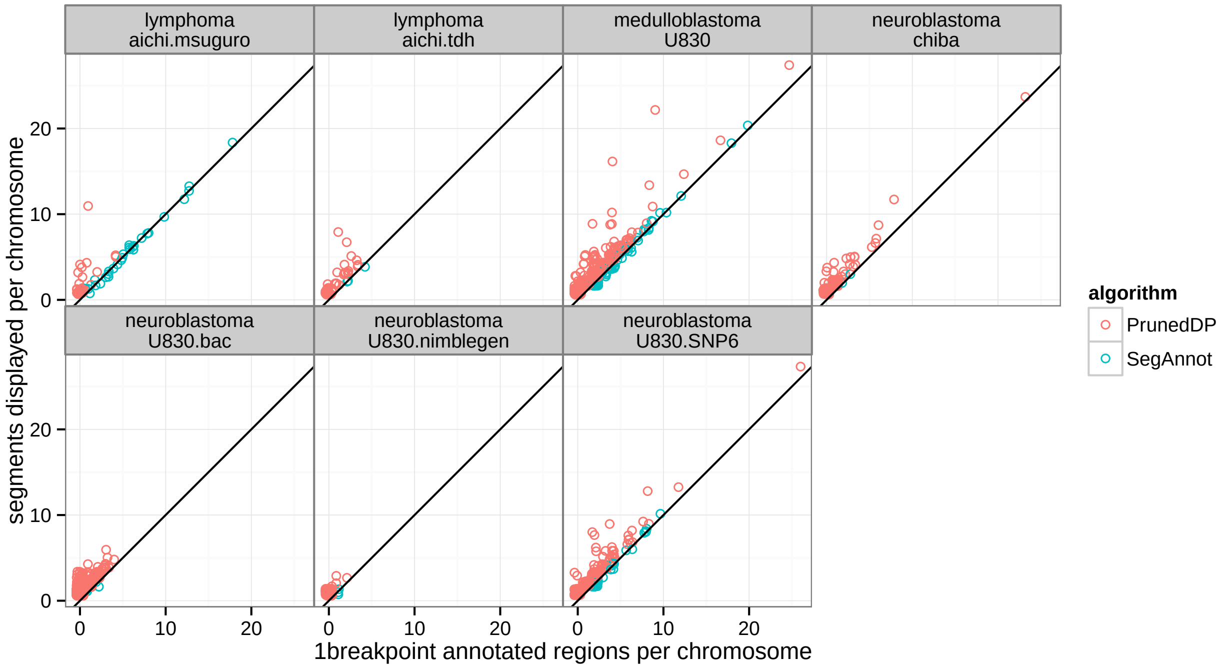

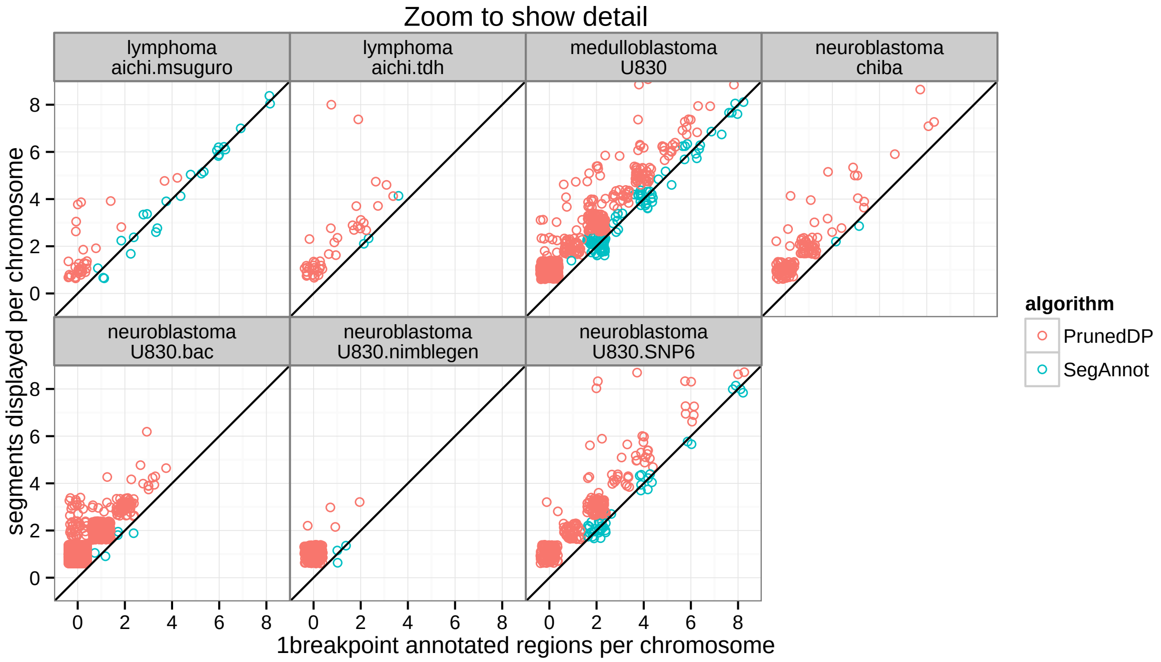

Figure S2: Copy number profiles of cell lines and tumor samples

In the figure below, the top 3 profiles come from neuroblastoma cell lines, and the bottom 3 profiles come from primary tumor samples (in the neuroblastoma.U830.bac data set). Both types of samples show about the same level of noise, so many breakpoints are visible and can be annotated.

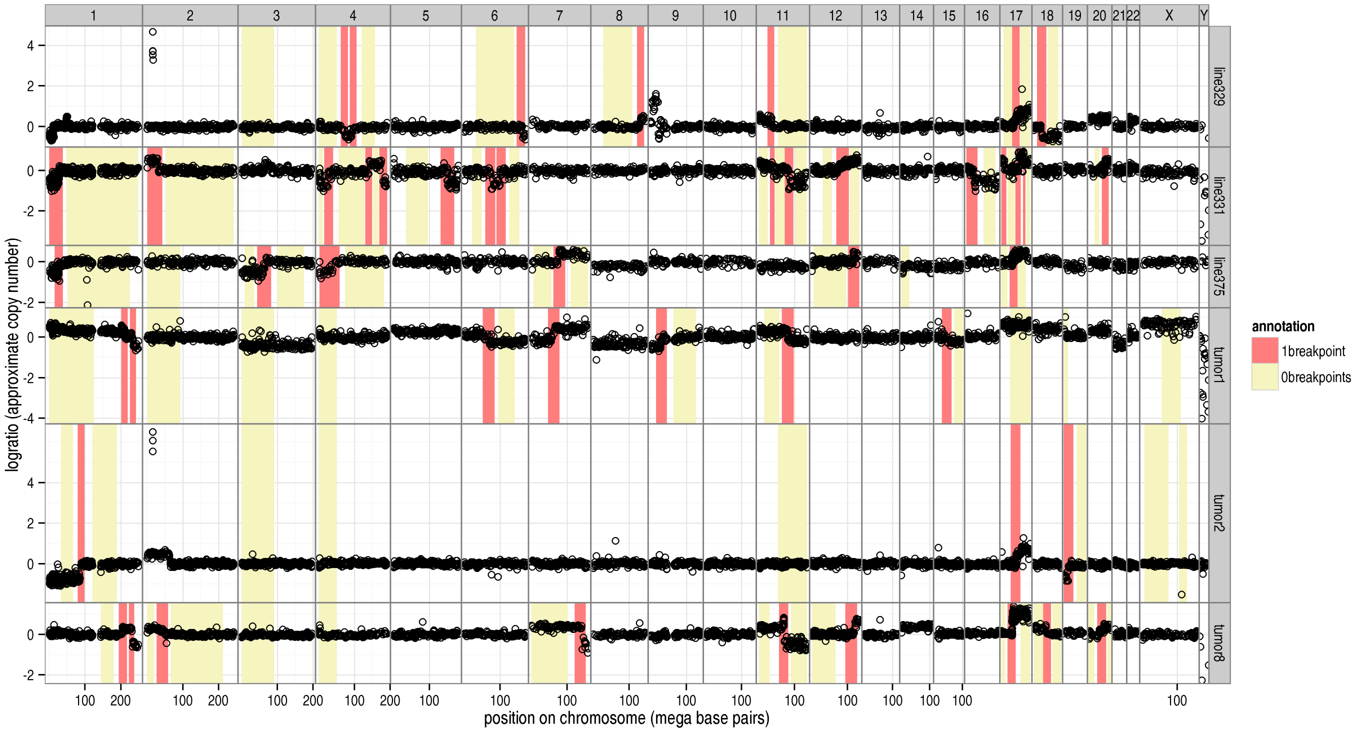

Figure S3: DNAcopy is slower than PrunedDP for larger profiles

The data we segmented were DNA copy number profiles from different types of arrays (BAC/PAC, Nimblegen, Affymetrix SNP6, etc). In total, there were 4467 chromosomes, with a range of 25 to 153,662 probes per chromosome.

For PrunedDP we ran `cghseg::segmeanCO` with `Kmax=52` (or lower if there were less than 52 probes). We report the total time it takes to calculate all 52 segmentation models.

We ran `DNAcopy::segment` using `undo.splits="sdundo"` and 31 different values of the `undo.SD` parameter, from 0 to 20. The other parameters were kept at default values. We report the total time it takes to calculate all 31 models (dnacopy.sd) and the time it takes to calculate just one model (dnacopy.default).

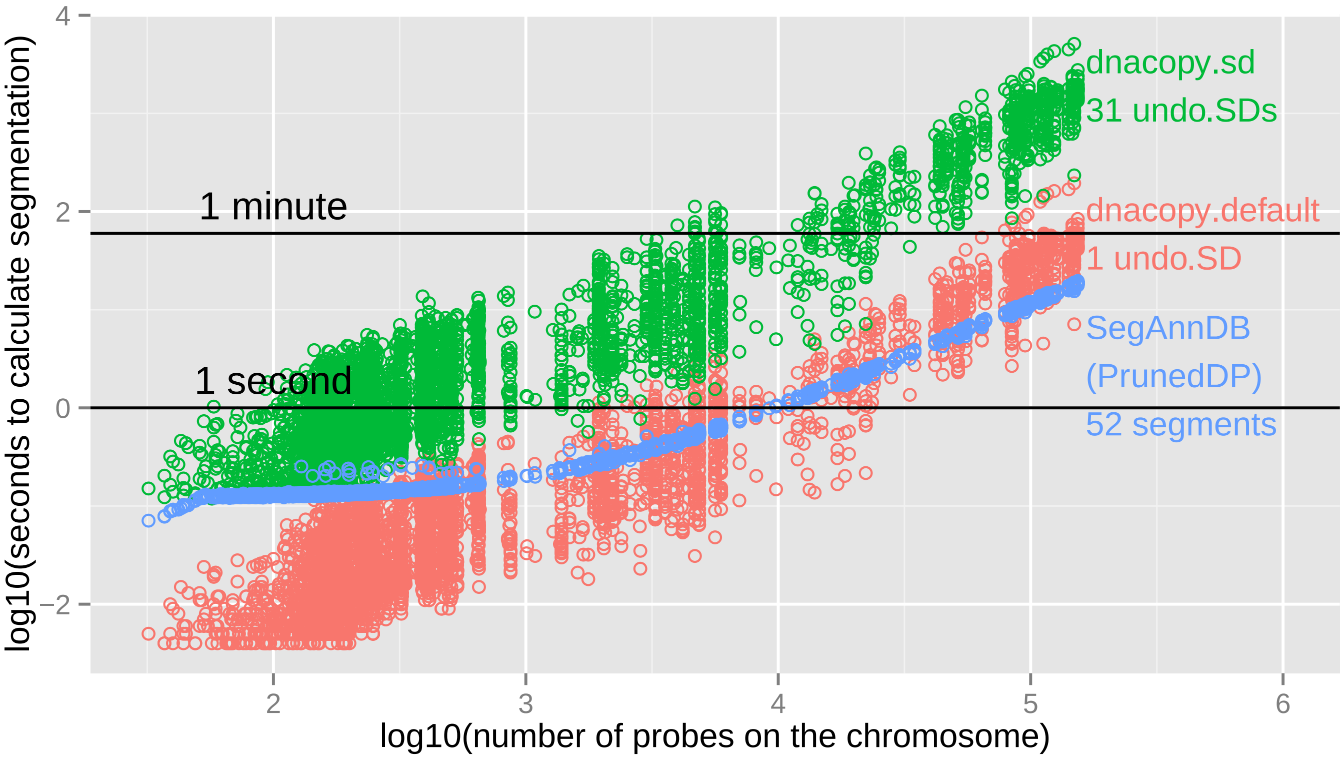

Figure S4: Profile 20104 chr2 has 26 breakpoint annotations

This chromosome had 26 regions annotated to have 1breakpoint (red rectangles), the most of any chromosomes we examined. Breakpoints detected by the SegAnnDB (PrunedDP) or dnacopy models are drawn in vertical dashed lines:

- **SegAnnDB (PrunedDP)** models with 26 and more breakpoints had 0 annotation error for these annotations of this chromosome. The model with 26 breakpoints is shown below.
- **dnacopy.default** detected 58 breakpoints on this chromosome, committing 2 false positives (more than 1 breakpoint detected in a region annotated to have only 1). Note that only 35 of these breakpoints are visible in the plot below, since we only plotted the probes in the middle of the chromosome (18,663 probes out of 153,662 total), to zoom in on the breakpoints and annotated regions.
- **dnacopy.sd** with undo.SD=1 (and defaults for other parameters) detected 42 breaks total and 30 breaks in the region displayed below, which gives 0 annotation errors.

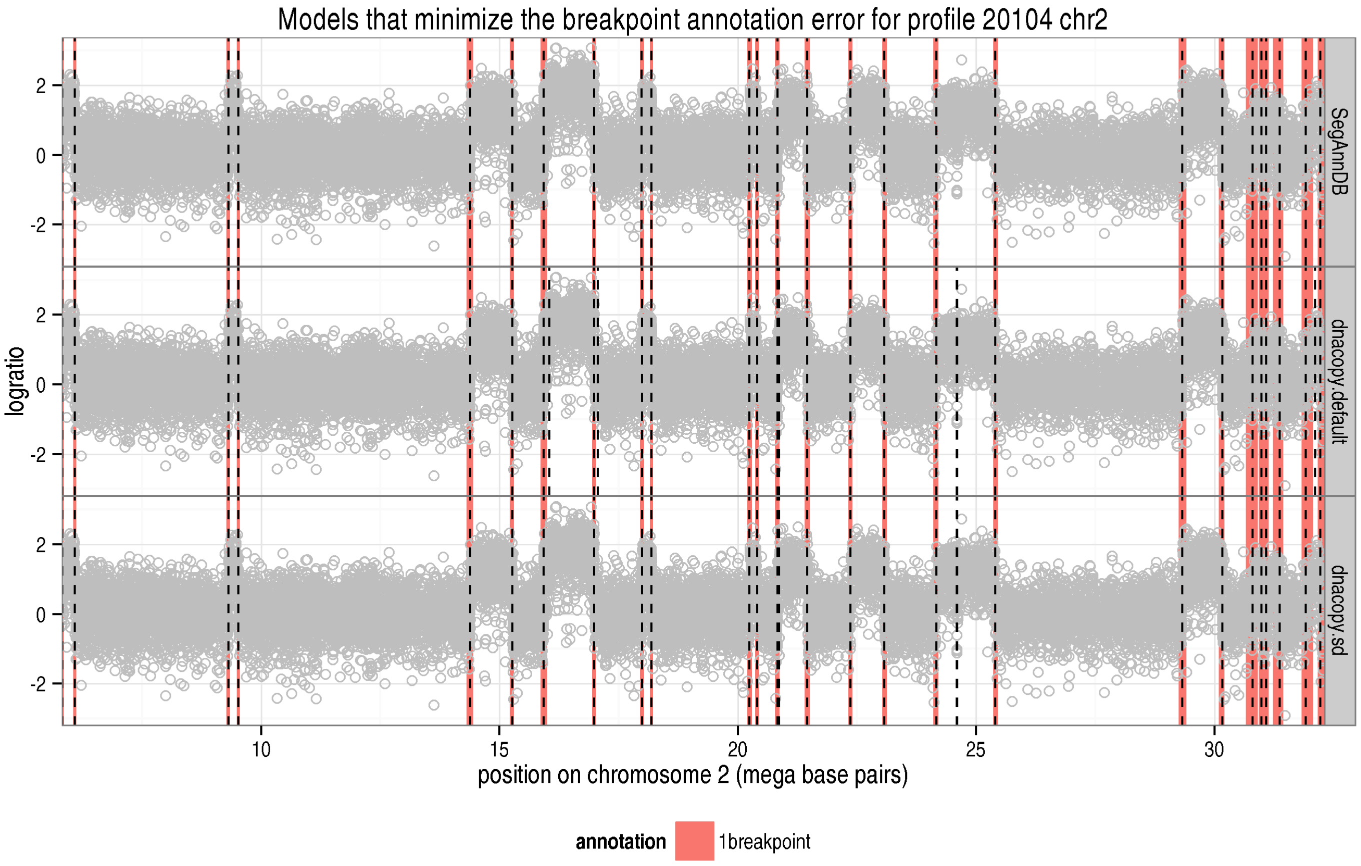

Figure S5: Test error of supervised models decreases as annotations are added

For each data set (panels), we compare test error of 3 models trained on $2, \ldots, 50$ annotated chromosomes (or less for the lymphoma data sets for which we had few annotations). Lines show the mean and shaded bands show the standard deviation over 60 randomly chosen training sets of the given size. It is clear that the unsupervised dnacopy.default algorithm does not use the annotated regions so does not improve its breakpoint detection as annotations are added. In contrast, for the supervised dnacopy.sd and SegAnnDB models, the test error decreases as annotations are added. Note the large test error of 20–60% for some methods, which motivates spending time on interactive annotation on SegAnnDB, which always achieves 0% training error (Table S6).

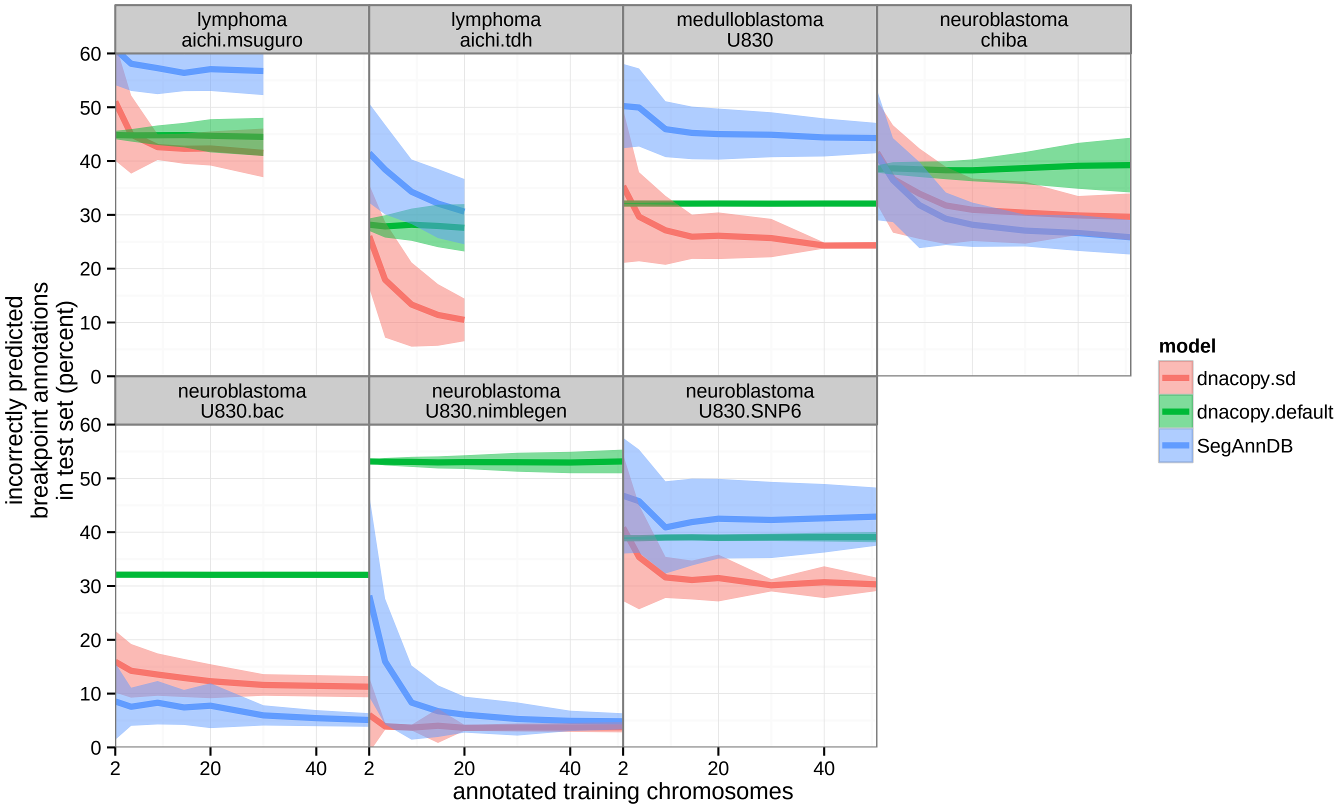

Figure S6: Annotated breakpoints increases with number of probes

We plot the number of annotated regions versus number of probes for each annotated chromosome. There is 1 row for each annotation data set, and 1 column for each annotation type (0 or 1 breakpoints). It is clear that the number of 0breakpoints annotations is constant as the number of probes increases. In contrast, the number of 1breakpoint annotations is larger for high density profiles, highlighting the fact that more time is sometimes necessary to annotate high density profiles.

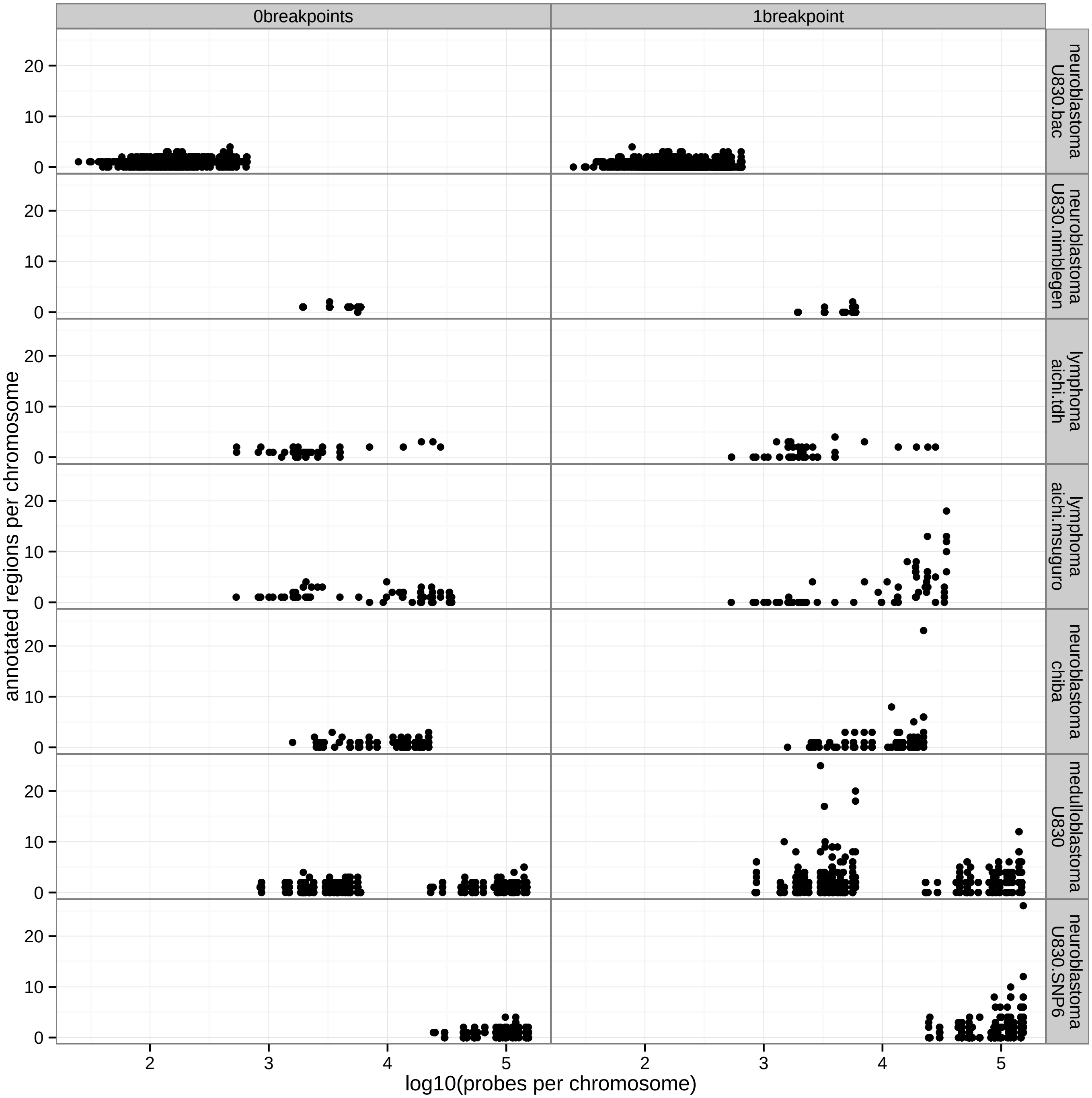

Figure S7: Two experts do not always label the same breakpoints

The 5 chromosomes below are lymphoma copy number profiles that were labeled by two different experts (msuguro and tdh). The breakpoint annotations are drawn as rectangles and the breakpoints detected by SegAnnDB are drawn as vertical black lines. Note that some red 1breakpoint annotations are too small to be clearly seen in this zoomed out plot. Nonetheless, it is clear that the two experts often have different definitions of relevant breakpoints.

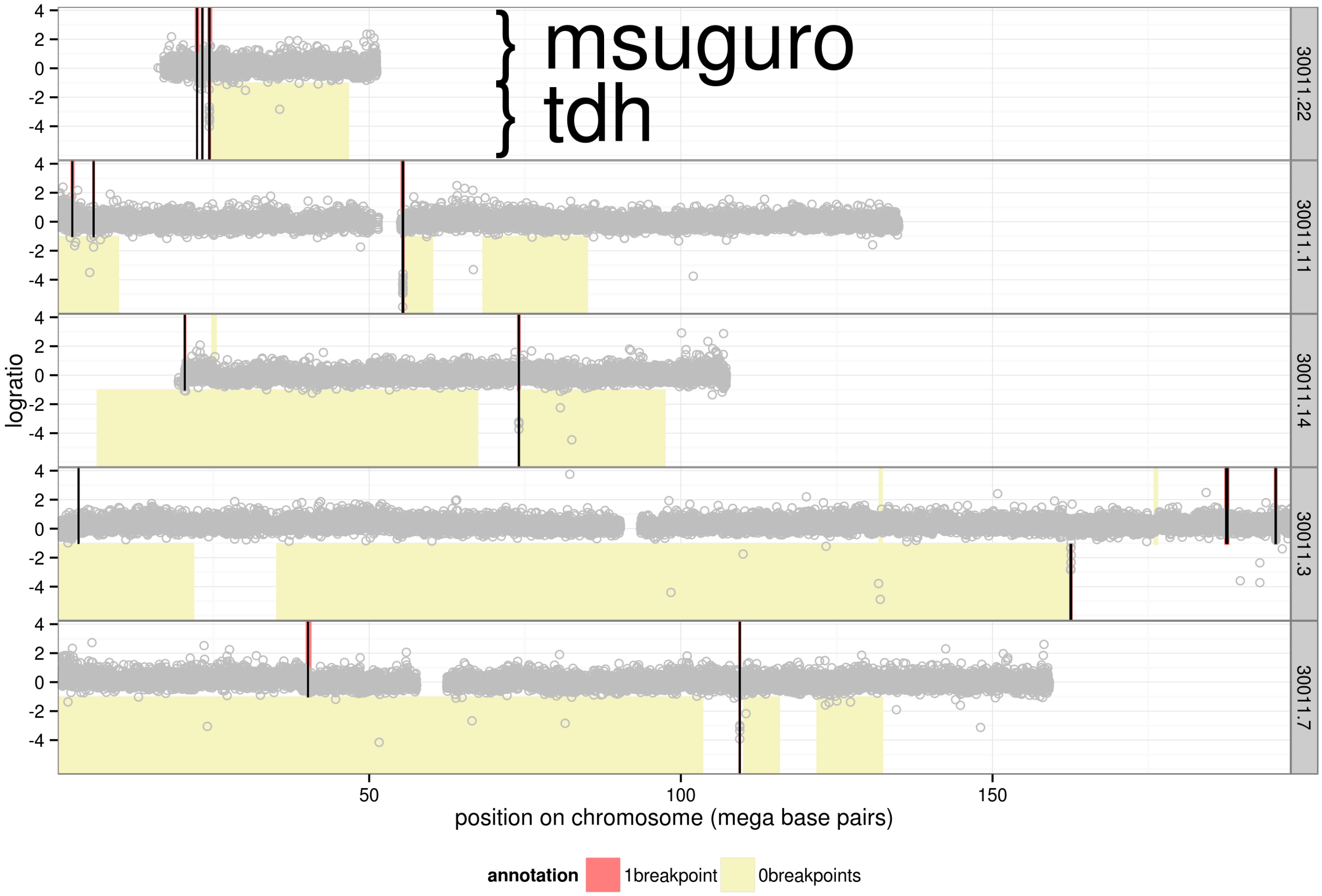

Supplement: Supplementary Data [file supp_btu072_HOCKING-supplementary.pdf]
